# Supplementary material for: Nodule Synthetic Bacterial Community as Legume Biofertilizer under Abiotic Stress in Estuarine Soils
Source: Plants (Basel). 2023 May 24;12(11):2083. doi: 10.3390/plants12112083 (PMC10255395; doi:10.3390/plants12112083)
Supplement: Supplementary file 1 [file plants-12-02083-s001.zip › plants-2385928-supplementary.pdf]

Supplementary Table S1. PGP properties, enzymatic activities and the maximum tolerable concentration of heavy metals of the bacterial strains [19].

PGP properties	N4	N8	N10	N12
Phosphate solubilization	11	12	15	14
Siderophore production	73	44	13	10.2
IAA production	1.562	17.939	1.266	1.000
Biofilm formation	1.826	1.104	0.476	0.308
ACC deaminase activity	9.677	9.987	–	–
N fixation	+	+	+	+
**Enzymatic activities**				
DNAse	–	–	+	+
Amylase	–	–	–	–
Cellulase	–	+	+	+
Lipase	–	–	–	–
Pectinase	–	–	–	–
Protease	+	+	–	–
Chitinase	–	–	–	–
**Maximum Tolerable Concentration**				
Cd	0.7	0.4	0.4	0.1
As	1	2.5	1.9	1.8
Cu	0.4	0.4	1.4	2.5
Zn	0.1	–	1.8	1.4

"+": presence of the activity; "–": absence of the activity; "nd": no determinated. Values of phosphate solubilization and siderophores production express the diameter of the halo in mm. Values of IAA production are expressed in $mg{\cdot}L^{-1}$. Values of ACC deaminase activity are expressed in µmoles α-ketobutyrate·mg protein$^{-1}{\cdot}h^{-1}$). Values of maximum tolerable concentration of heavy metals are expressed in mM. N4: *Pseudomonas* sp. N4, N8: *Pseudomonas* sp. N8, N10: *Ensifer* sp. N10, and N12: *Ensifer* sp. N12.

Supplementary Table S2. Mineral salts concentrations in *M. sativa* plant tissues inoculated with different inoculums after 60 days in pots under greenhouse conditions using sterilized soil from the upper marshes of the Odiel river supplemented with 60 mM of NaCl as substrate.

Treatments	Ca (g/100g)	Fe (mg/Kg)	K (g/100g)	Mg (g/100g)	Mn (mg/Kg)	Na (g/100g)	P (g/100g)
**Shoot**							
**C-**	3.435 ± 0.010[c]	278.851 ± 0.012[c]	1.156 ± 0.005[b]	0.353 ± 0.012[c]	130.216 ± 0.010[a]	0.439 ± 0.000[a]	0.082 ± 0.004[a]
**N10**	2.781 ± 0.006[b]	263.215 ± 0.010[b]	1.026 ± 0.010[a]	0.291 ± 0.010[b]	210.924 ± 0.013[b]	0.511 ± 0.001[b]	0.159 ± 0.005[b]
**CSN**	1.734 ± 0.008[a]	228.344 ± 0.008[a]	1.578 ± 0.004[c]	0.223 ± 0.010[a]	333.276 ± 0.015[c]	0.640 ± 0.014[c]	0.352 ± 0.001[c]
**Roots**							
**C-**	0.487 ± 0.015[b]	922.526 ± 0.065[c]	0.650 ± 0.007[a]	0.536 ± 0.010[c]	140.342 ± 0.028[a]	0.501 ± 0.006[a]	0.081 ± 0.001[a]
**N10**	0.505 ± 0.016[c]	862.374 ± 0.064[a]	0.633 ± 0.006[a]	0.364 ± 0.014[b]	350.852 ± 0.020[b]	0.687 ± 0.005[b]	0.144 ± 0.000[b]
**CSN**	0.455 ± 0.021[a]	889.589 ± 0.045[b]	0.817 ± 0.005[b]	0.216 ± 0.011[a]	797.661 ± 0.031[c]	0.811 ± 0.007[c]	0.349 ± 0.001[c]

Values are mean ± S.D. (n=2). Different letters indicate means that are significantly different from each other (one-way ANOVA; LSD test, $P < 0.0001$). C-: non inoculation, N10: inoculation with *Ensifer* sp. N10, CSN: Co inoculation with *Pseudomonas* sp. N4, *Pseudomonas* sp. N8, *Ensife*r sp. N10 and *Ensifer* sp. N12.
